# Design and application of artificial rare L-lysine codons in *Corynebacterium glutamicum*

**DOI:** 10.3389/fbioe.2023.1194511

**Published:** 2023-05-30

**Authors:** Cuiping Yang, Zehao Peng, Lu Yang, Bowen Du, Chuanzhuang Guo, Songsen Sui, Jianbin Wang, Junlin Li, Junqing Wang, Nan Li

**Affiliations:** ^1^ Department of Biological Engineering, Qilu University of Technology, Jinan, China; ^2^ Zhucheng Dongxiao Biotechnology Co., Ltd., Zhucheng, China; ^3^ State Key Laboratory of Biobased Material and Green Papermaking (LBMP), Qilu University of Technology, Jinan, China; ^4^ College of Biotechnology, Tianjin University of Science and Technology, Tianjin, China

**Keywords:** *Corynebacterium glutamicum*, gene knockout, rare codon, L-lysine, high flux

## Abstract

**Background:** L-lysine is widely used in the feed, food, and pharmaceutical industries, and screening for high L-lysine-producing strains has become a key goal for the industry.

**Methods:** We constructed the rare L-lysine codon AAA by corresponding tRNA promoter replacement in *C. glutamicum*. Additionally, a screening marker related to the intracellular L-lysine content was constructed by converting all L-lysine codons of enhanced green fluorescent protein (EGFP) into the artificial rare codon AAA. The artificial EGFP was then ligated into pEC-XK99E and transformed into competent *Corynebacterium glutamicum* 23604 cells with the rare L-lysine codon. After atmospheric and room-temperature plasma mutation and induction culture, 55 mutants (0.01% of total cells) with stronger fluorescence were sorted using flow cytometry, and further screened by fermentation in a 96-deep-well plate and 500 mL shaker.

**Results:** The fermentation results showed that the L-lysine production was increased by up to 9.7% in the mutant strains with higher fluorescence intensities, and that the highest screening positive rate was 69%, compared with that in the wild-type strain.

**Conclusion:** The application of artificially constructed rare codons in this study represents an efficient, accurate, and simple method for screening other amino acid-producing microorganisms.

## 1 Introduction

L-lysine, one of the eight essential amino acids involved in human metabolic balance, is involved in protein synthesis and in energy and fat metabolism. It is widely used in the feed, food, and pharmaceutical industries (Liu et al., 2022). L-lysine has entered the market as an important food additive due to its positive contribution to growth performance and protein deposition in pig, poultry, and fish tissues (Eggeling and Bott, 2015; Hamid et al., 2016; Nguyen and Davis, 2016). To meet the feed quality requirements of the continuously developing animal husbandry industry, 90% of L-lysine products are poured into the feed industry to improve feed quality (Felix et al., 2019). In addition, lysine has shown potential as a feedstock for the production of other high-value chemicals for active pharmaceutical ingredients, drugs, or materials (Cheng et al., 2018). Due to the significant nutritional and commercial value of L-lysine, its market demand continues to grow steadily.

Depending on the raw materials and production principles, L-lysine can be produced using the following three broad methods: protein hydrolysis, chemical synthesis, and microbial fermentation (Nakamori, 2017; Xu et al., 2019). However, microbial fermentation is currently the only method for the industrial production of L-lysine. *Corynebacterium glutamicum* and its mutant subspecies were the early strains applied for the industrial production of L-lysine (Xiao et al., 2020). To date, mainly modified strains of *Corynebacterium glutamicum* and *Escherichia coli* have been used for L-lysine industrial production (Li et al., 2017). The microbial fermentation method utilizes amino acid nutrient-deficient and amino acid structural analogue-resistant mutant strains that dismantle the feedback blocking and inhibitory effects of L-lysine. Both types of mutant strains achieve excessive accumulation of L-lysine for L-lysine production by altering the intracellular carbon source distribution (Wang et al., 2021). Although microbial fermentation has unique advantages over physical and chemical methods for L-lysine production, there has been a lack of effective means for high-throughput screening of high-yielding strains.

Most of the strains currently widely used for the industrial production of L-lysine are obtained by mutagenesis breeding methods, screening for nutrient-deficient strains, or using L-lysine structural analogues for strains treated with mutagenesis (Ikeda, 2017). Although conventional mutagenesis breeding can yield L-lysine-overproducing bacteria, traditional mutagenesis breeding uses nitrosoguanidine (NTG) and ultraviolet treatment (UV) for random mutation, these methods still suffer from low mutation efficiency and safety issues. In recent years, atmospheric and room-temperature plasma (ARTP) mutagenesis has been widely used, with the characteristics of rapid mutation, high efficiency and high safety (Zhang et al., 2014). Therefore, ARTP may be an effective mutagenesis tool to increase the yield of L-lysine. Structural analogues may interfere with normal cell growth, and mutants may not survive the side effects. Currently, the screening of L-lysine high-yielding strains using the L-lysine structural analogue aminoethylcysteine after physical or chemical mutagenesis does not provide effective enhancement of L-lysine production. As a result, there is a need for high-throughput methods for screening high-yielding amino acid-producing strains that combine accuracy and sensitivity and can be widely applied to different species of microbial cells.

In recent years, strategies for screening high-yielding amino acid-producing strains using genes rich in rare codons have been proposed. Due to the degeneracy of codons, the same amino acid can be encoded by many different codons, and the decoding rates of synonymous codons vary widely, depending mainly on the abundance of homologous tRNA (Yu et al., 2015; Guimaraes et al., 2020). Common codons are recognized by rich tRNA and are translated more efficiently than rare codons, especially under amino acid starvation conditions, where the charge level of rare isoreceptors is immediately close to zero while the charge level of common isoreceptors remains high for several minutes (Dittmar et al., 2005; Subramaniam et al., 2014). Thus, contrary to “codon optimization”, we can reduce the expression of heterologous proteins in the host by replacing common codons with synonymous rare codons. Furthermore, for genes with rare codons, if the amount of amino acids in the cell is sufficient to support rare tRNA charging, their translation can be maintained under amino acid starvation conditions, and these strains are likely to be amino acid high-yielding strains (Zheng et al., 2018). Based on the above strategy, in this study, the rare lysine codon AAA was artificially constructed by replacing the promoter of the lysine tRNA gene (anticodon UUU), and the link between the intracellular L-lysine concentration and fluorescence intensity was established. L-lysine high-yielding strains were obtained by combining ARTP mutagenesis and high-throughput fluorescence-activated cell sorting (FACS) screening. The application of artificially constructed rare codons in this study provides an efficient, accurate, and simple screening method for a broader spectrum of microbial cultures.

## 2 Materials and methods

### 2.1 Bacterial strain plasmids and culture media

All the strains and plasmids used in this study are listed in Table 1. *Escherichia coli* DH5α and BL21 (DE3) cells were grown in Luria-Bertani (LB) medium containing 10 g/L peptone, 5 g/L yeast extract, and 10 g/L NaCl at 37°C and pH 7.2. *Corynebacterium glutamicum* was cultured at 30°C and pH 7.0. The seed medium LB + glucose (LBG) contained 10 g/L peptone, 5 g/L yeast extract, 10 g/L NaCl, and 5 g/L glucose. The recombinant colony was grown on LBHIS AGAR medium containing 5 g/L peptone, 5 g/L NaCl, 2.5 g/L yeast extract, 2.5 g/L glucose, 18.5 g/L brain-heart extract, and 91 g/L sorbitol. The fermentation medium contained 20 g/L glucose, 30 g/L corn pulp, 20 g/L (NH_4_)_2_SO_4_, 10 g/L CH_3_COONa, 5 g/L urea, 1.34 g/L L-alanine, 2 g/L KH_2_PO_4_, 1.35 g/L MgSO_4_·7H_2_O, 0.02 g/L FeSO_4_·7H_2_O, 0.04 g/L MnSO_4_·H_2_O, 0.008 g/L nicotinamide, 0.001 g/L biotin, and 0.04 g/L L-leucine. *Corynebacterium glutamicum* strains are usually grown in medium supplemented with 25 μg/mL kanamycin (50 μg/mL for *E. coli*).

**TABLE 1:** Strains and plasmids used in this study.

Strain/plasmid	Genetic characteristics	Source
**Strains**		
*Corynebacterium glutamicum* 23604	Wild-type	CICC
*Escherichia coli* DH5α	*Δ*(*lacZYA -argF*)*U169, deoR, recA1, endA1, hsdR17, supE44, gyrA96, relA1*	Vazyme
*E. coli* BL21 (DE3)	*λ*(*DE3[lacI lacUV5-T7 gene1 ind1 sam7 nin5]*)*, Δ(ompT-nfrA)885*	Vazyme
*C. glutamicum-Parg*	CICC 23604, contains the promoter Parg	This work
*C. glutamicum-Pgit*	CICC 23604, contains the promoter Pgit	This work
**Plasmids**		
pK19mobsacB	allows for selection of double crossover *C. glutamicum*	
pK19mobsacB-tRNA	pK19mobsacB containing internal deletion of 1,044 bp fragment of *Lysine-tRNA*	This work
pK19mobsacB-*Parg*	pK19mobsacB containing a 1741-bp fragment of *Parg*	This work
pK19mobsacB-*Pgit*	pK19mobsacB containing a 1325-bp fragment of *Pgit*	This work
pEC-XK99E	shuttle expression vector containing the IPTG inducible Ptrc promoter	
pEC-XK99E-*egfp* ^*M*^	pEC-XK99E containing *egfp* ^*M*^	This work
pEC-XK99E-*ebfp* ^*M*^	pEC-XK99E containing *ebfp* ^*M*^	This work
pEC-XK99E-*mCherry* ^*M*^	pEC-XK99E containing *mCherry* ^*M*^	This work

IPTG, isopropyl ß-D-1-thiogalactopyranoside; *egfp* ^*M*^, enhanced green fluorescent protein; *ebfp* ^*M*^ *, enhanced blue fluorescent protein*.

### 2.2 Genome sequence and codon usage frequency analysis of *Corynebacterium glutamicum* 23604

Illumina MiSeq (https://www.illumina.com.cn) and PacBio RSII (Pacific Biosciences, United States) were used to sequence the genome of *C. glutamicum* 23604 following gene element prediction of the whole genome sequence (Piwowarek et al., 2020).

### 2.3 Construction of promoter replacement vectors

pK19mobsacB-*tRNA* was constructed using the following method. The genome of *C. glutamicum* 23604 was extracted using the Ezup Column Bacteria Genomic DNA Purification Kit (Sangon Biotech, Shanghai, China). The polymerase chain reaction (PCR) primers used in this study are listed in Table 2. In the first round of PCR, the upstream and downstream regions of the lysine tRNA UUU gene were amplified using primer pairs P1/P2 and P3/P4, with *C. glutamicum* 23604 genomic DNA as the template. In the second round of PCR, the product of the first round of PCR was used as the template, and P1/P4 was used as the primer pair. The first round of PCR conditions were as follows: pre-degeneration at 95°C for 3 min, 30 cycles of degeneration at 95°C for 30 s, 55°C for 30 s, 72°C for 45 s, followed by extension at 72°C for 10 min and holding at 4°C. The second round of PCR conditions were as follows: (1) pre-degeneration at 95°C for 3 min, five cycles of degeneration at 95°C for 30 s, 54°C for 30 s, and pre-extension at 72°C for 90 s, followed by extension at 72°C for 2 min; (2) pre-degeneration at 95°C for 3 min, 30 cycles of degeneration at 95°C for 30 s, 54°C for 30 s, 72°C for 1.5 min, followed by extension at 72°C for 10 min and holding at 4°C. Fragments of a fusion gene, namely, a 1,044 bp fragment of lysine tRNA, were thus obtained. The plasmid pK19mobsacB was digested with the restriction enzymes *Eco*RI and *Hin*dIII (Thermo Fisher Scientific, United States). Seamless cloning was performed using a One Step Cloning Kit (Vazyme Biotech, Nanjing, China), and then the recombinant plasmids were transfected into competent *E. coli* DH5α cells. Finally, positive recombinant strains were screened by colony PCR, and DNA sequencing was performed.

**TABLE 2:** Primers used in this study.

Primer	Sequence (5′→3′)	Restriction site
P1	tat​gac​cat​gat​tac​aag​ctt​CGG​TGA​AAA​CCT​GAA​CAG​TGC	*Hin*dIII
P2	TGG​TCT​GAG​TTG​GGC​CTA​TAG​ACC​CCT​GTT​TTG​GAG​AAT​GCT	
P3	AGC​ATT​CTC​CAA​AAC​AGG​GGT​CTA​TAG​GCC​CAA​CTC​AGA​CCA	
P4	acg​acg​gcc​agt​gcc​gaa​ttc​TAC​ATC​TCC​AGC​TTC​ATC​ACC​CC	*Eco*RI

The P*git* inducible promoter and arginine rare tRNA UCU P*arg* promoter sequences in the genome of *C. glutamicum* were found in the National Centre for Biotechnology Information (NCBI) database (https://www.ncbi.nlm.nih.gov/), and the upstream and downstream homologous arm sequences required for homologous double exchange were added to both sides of the sequences. The L-P*git*-R and L-P*arg*-R gene fragments (GenScript Biotech, Nanjing, China) were obtained and cloned into the *Eco*RI-and *Hin*dIII-digested pK19mobsacB plasmid sites, respectively. The recombinant plasmids were then transfected into competent *E. coli* DH5α cells. Finally, colony PCR was used to screen for positive clones, and the identity of the fusion fragments was confirmed by sequencing.

### 2.4 Transformation and isolation of engineered strains

In advance, competent *C. glutamicum* 23604 cells were prepared (Xiao et al., 2020). The competent cells were then given a 10 μL aliquot of DNA before being transferred to a 2 mm electroporation cuvette (Bio-Rad Laboratories, Hercules, California, United States), with the parameters set at 2.2 kV and 5 ms. Following the electroporation, a 6-min heat shock was administered. Then, the plates were incubated for 2 h at 30°C and plated on LBHIS AGAR medium containing 25 μg/mL kanamycin for 1 day.

Two rounds of forward selection for homologous recombination were performed. Selection was first performed under kanamycin resistance conditions to integrate the plasmid into the chromosome. Strains that grew normally under kanamycin resistance conditions were inoculated in LB medium containing 15% sucrose (LBS). After three passes, they were inoculated in LB AGAR medium containing kanamycin or LB AGAR medium containing sucrose, respectively. Strains that grew only on LB AGAR medium containing sucrose were selected. Using the genome as a template, PCR amplification with P1/P4 primers was performed to identify clones carrying the required deletions or undergoing allele exchange. When the lysine tRNA gene was knocked out, the strains that were screened by the second round of sucrose (15%) could not be obtained. P*git* and P*arg* promoter replacements amplified DNA fragments of 1741 bp and 1,325 bp, respectively.

### 2.5 Determination of promoter substitution effect

To determine the expression of tRNA-UUU, RT-qPCR was performed using a fluorescent quantitative PCR instrument (Applied Biosystems, United States) to analyse the expression of tRNA-UUU in wild-type and promoter replacement strains, and the 16s rRNA gene was selected as an internal reference gene for quantification. The experimental methods are shown in the Supplementary Material.

Wild-type strain *C. glutamicum* 23604 and three colonies of the promoter replacement strain were inoculated into liquid LBG medium and cultured overnight at 200 r/min and 30°C. Sixteen hours later, the seed solution was inoculated in 100 mL fermentation medium at 10%. During the fermentation process, samples were taken from the fermentation medium every 12 h to measure the OD_600_ value and L-lysine concentration. More than three experiments were performed, and the average OD_600_ and L-lysine concentration values were calculated.

### 2.6 Construction and transformation of a fluorescent protein expression vector

To construct pEC-XK99E-enhanced green fluorescent protein (egfpM), pEC-XK99E-enhanced blue fluorescent protein (ebfpM), and pEC-XK99E-mCherryM, the lysine codons in the egfp, ebfp, and mCherry genes were all replaced by the AAA codon. EgfpM, ebfpM, and mCherryM were obtained by gene fragment synthesis (Genscript Biotech, Nanjing, China) and cloned into pEC-XK99E digested with the restriction enzyme EcoRI (Thermo Fisher Scientific, United States) for the expression of different fluorescent proteins. The recombinant plasmids were then transfected into *E. coli* DH5α competent cells. Finally, positive recombinant strains were identified using colony PCR and DNA sequencing.

### 2.7 Expression of fluorescent protein

Three fluorescent protein granules were extracted and transformed into the preprepared C. glutamicum 23604 receptor cells. The recombinant strains obtained were activated and inoculated into 100 mL LBG medium containing 25 μg/mL kanamycin with an initial OD600 of 0.3. The cells were placed in an incubator with constant temperature oscillation at 30°C and a speed of 200 r/min. After 24 h of culture, the expression of fluorescent protein was induced. Finally, fluorescence intensity was measured using a Biotek Microplate Reader (Biotek Instruments, Inc., Winooski, VA, United States).

### 2.8 Fluorescence screening system constructed based on the artificial rare codon

pEC-XK99E-*egfp* ^*M*^ was transformed into the strains that replaced the promoter, and the positive recombinant colonies were selected and inoculated into the LBG medium containing 25 μg/mL kanamycin and incubated at 30°C and 200 r/min. Induced expression was performed according to the optimization results of the single factor experiment.

### 2.9 ARTP mutagenesis and flow cytometry screening

Compared with traditional mutagenesis, the ARTP mutation system can generate a larger gene mutation library. First, the two recombinant strains containing the EGFP^*M*^ screening label were cultured in LBG medium containing 25 μg/mL kanamycin at an OD_600_ of 0.6–0.9, then coated with 10 μL medium uniformly on the stainless-steel minidisc. After exposure to ARTP for 1, 3, 5, 7, and 9 min, the fatality rate was calculated. The optimal ARTP mutants with over 90% treatment lethality were selected and washed with 1 mL LBG medium. Then, 500 μL was inoculated in 50 mL LBG medium and cultured at 30 min and 200 r/min until the OD_600_ reached 1.0. Isopropyl *β*-D-1-thiogalactopyranoside (IPTG) with a final concentration of 0.6 mM was added to the medium and incubated at 30°C for 10 h. The 1 mL culture medium was diluted to an OD_600_ of 1.0, and ARTP mutants with strong fluorescence were sorted using flow cytometry under the sort condition of green fluorescence (Mohsin et al., 2015). The cultured cells were washed and resuspended in a potassium phosphate buffer. Then, the cells were analysed using FACS (Beckman Coulter MoFlo XDP, United States), with an excitation line at 488 nm, fluorescence detection at 535 nm, and a sample pressure of 60 psi. The nozzle diameter was set to 70 μm. Sterile, filtered phosphate buffered saline was used as the sheath solution. Data analysis was performed using the Beckman Summit 5.2 software. In the mutant library selection, a gate containing 0.01% of total cells was set up according to the pre-analysis of the mutant library, and cells with higher expression were collected and further fermented in a 96-well plate.

### 2.10 Fermentation of mutant *Corynebacterium glutamicum* strains

The screened mutants were inoculated into 96-well plates and cultured in LBG medium for 24 h as the seed solution. Meanwhile, *C. glutamicum* 23604 was inoculated into 96-well plates containing LBG medium using flow cytometry. Seeds of wild-type *C. glutamicum* 23604 and ARTP mutants were inoculated at 5% into 96-deep-well plates filled with fermentation medium and fermented at 30°C and 600 r/min for 48 h. The content of L-lysine in the fermentation broth was measured at 24 h and 48 h using a biosensor analyzer SBA-40E (Institute of Biology, Shandong Academy of Sciences, Shandong, China) (Geng et al., 2013). The mutant and wild-type strains with the highest L-lysine production obtained in 96-well plates were inoculated in liquid LBG medium to establish three parallel experiments and incubated overnight at 200 r/min and 30°C. After 16 h, the seed solution was inoculated into 100 mL of fermentation medium at 10% inoculum. Following 24 h of fermentation, samples of the fermentation medium were collected at 12 h intervals, and OD_600_ values, as well as L-lysine concentrations, were measured. The average OD_600_ and L-lysine concentration values of more than three experiments were calculated.

### 2.11 Statistical analysis

Statistical analysis was performed using IBM SPSS Statistics 29 (IBM SPSS, Turkey). One-way ANOVA was used to analyse the significance of mean difference of samples. The significance of differences was estimated with 0.05 level of confidence.

## 3 Results

### 3.1 Genome sequencing and amino acid codon analysis

**FIGURE 1:**
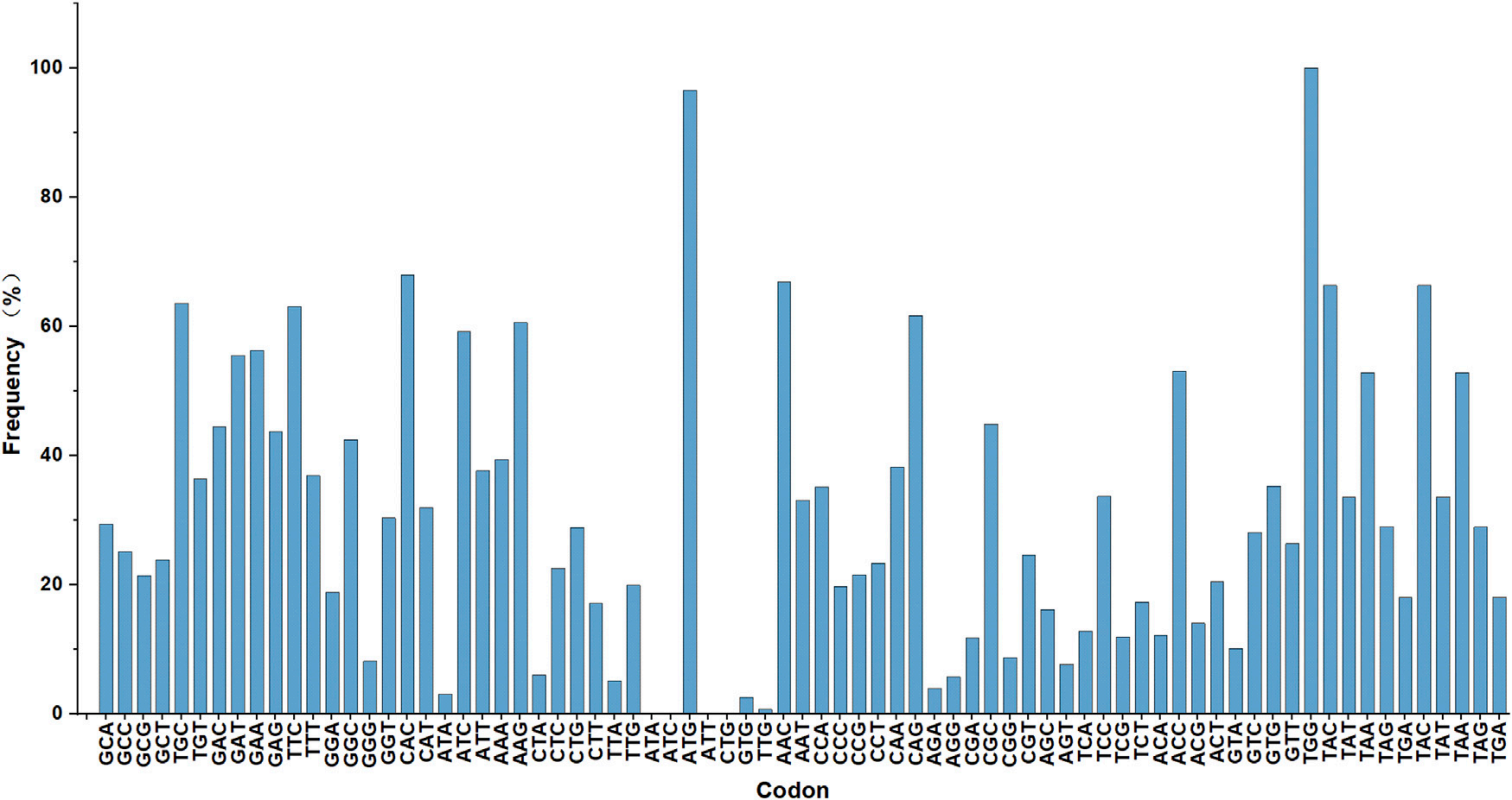
Genome sequencing analysis. The frequency distribution of amino acid codon usage in the *Corynebacterium glutamicum* 23604 genome.

**FIGURE 2:**
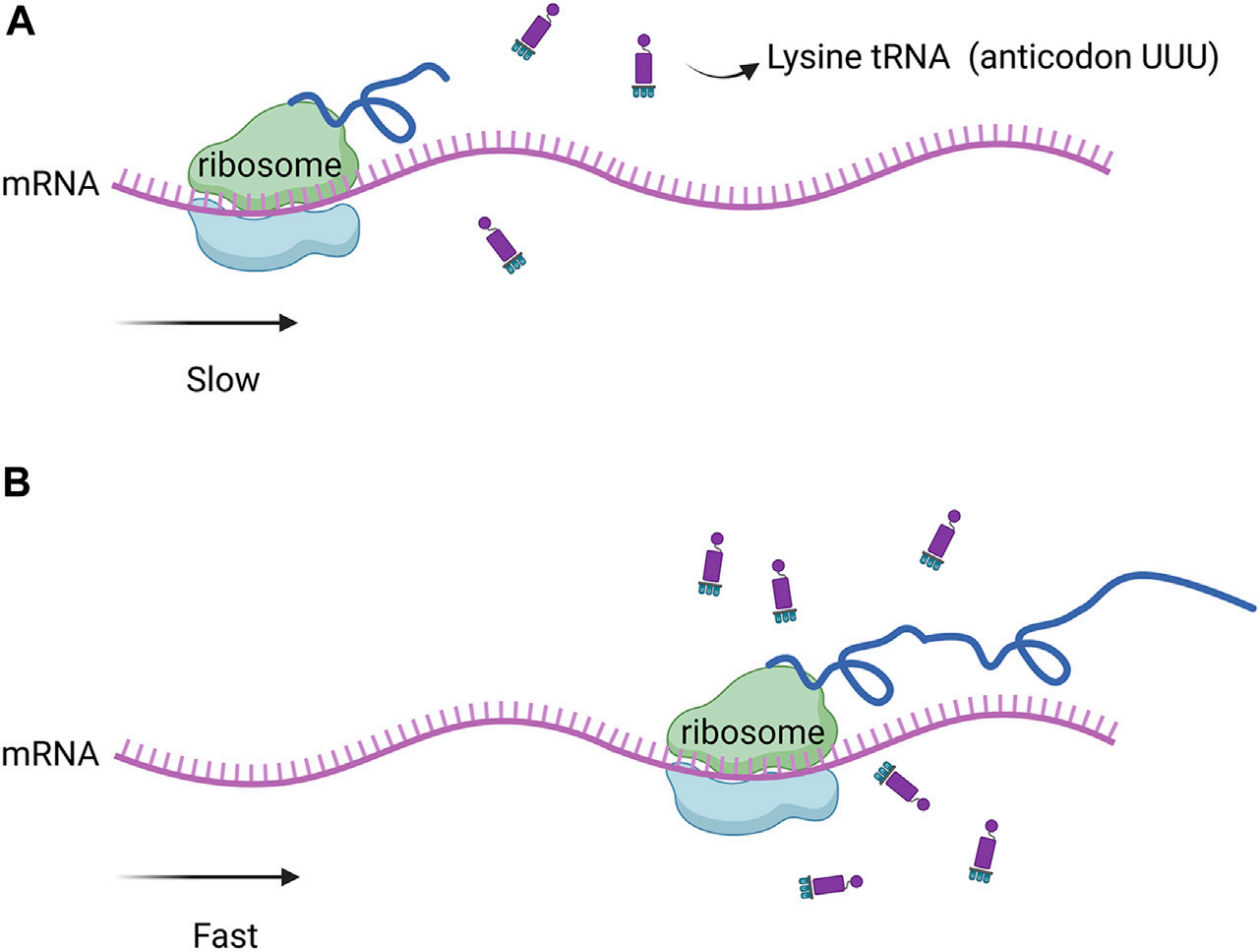
**(A)** Low abundance of rare tRNA significantly slowed down protein expression. **(B)** when the concentration of amino acids in the cell increased, the recognition of the rare codon of tRNA was bound to the corresponding amino acid by amino tRNA synthase, the expression of genes containing the rare codon was restored.

Genome sequencing and analysis showed that some codons were rare codons of *Corynebacterium glutamate* (Figure 1). For example, in the genome of *C. glutamicum* 23604, six codons encoded arginine, among which AGA was used less than 4%, while two codons, AAA and AAG, encoded lysine, with a frequency distribution of 40% and 60%, respectively. There was only one tRNA with the anticodon UUU, and two tRNAs with the anticodon CUU, all of which were single-copy genes. In addition, the frequency of the natural rare arginine codon AGA was very low, only 4%. Therefore, there was no natural rare L-lysine codon in *C. glutamicum* 23604. Consequently, this codon cannot be used for the screening of L-lysine high-yielding strains. In this study, the strategy of artificial construction of the rare codon was adopted. The translation of the rare codon is affected by the corresponding rare tRNA, and the regulation of the relevant translation level is particularly significant when the corresponding amino acid is deficient in cells. However, when the concentration of amino acids in the cell is increased to allow the rare codon recognition of tRNA to bind to the corresponding amino acid under the action of aminoyl tRNA synthase, genes containing the rare codon can be translated and expressed (Figure 2). In this study, tRNA with the UUU anticodon was constructed to enable the recognition of the rare artificial lysine codon AAA.

### 3.2 Effect of L-lysine tRNA knockout on *Corynebacterium glutamicum*

Plasmid pK19mobsacB was used to construct the knockout vector, and tRNA deletion strains with anticodon UUU were expected to be obtained through double exchange homologous recombination. The positive strain from the first round of homologous recombination was screened using kanamycin. The strains that successfully underwent the first homologous recombination were inoculated in LBG medium for culture, and then coated on LBS AGAR medium after gradient dilution. None of the strains grew, so the strains that underwent the second homologous recombination failed to be successfully screened. The results showed that *C. glutamicum* 23604 cannot be dependent on a tRNA whose only codon is CUU.

### 3.3 Effect of tRNA promoter replacement on *Corynebacterium glutamicum*

**FIGURE 3:**
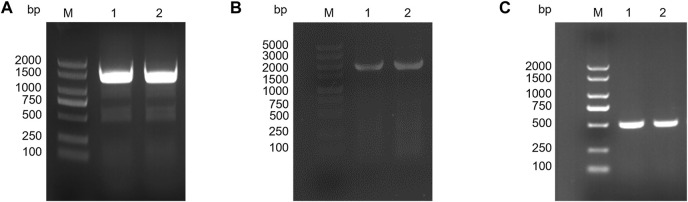
Gel electrophoresis of PCR products. **(A)** PCR gel showing the expected product insert size of 1,325 bp for the L-P*arg*-R. M, DL2000 DNA marker. **(B)** PCR gel showing the expected product insert size of 1741 bp for L-P*git*-R. M, DL5000 DNA marker. **(C)** PCR gel showing the expected product insert size of 548 bp for *egfp* ^*M*^. M, DL2000 DNA marker.

**FIGURE 4:**
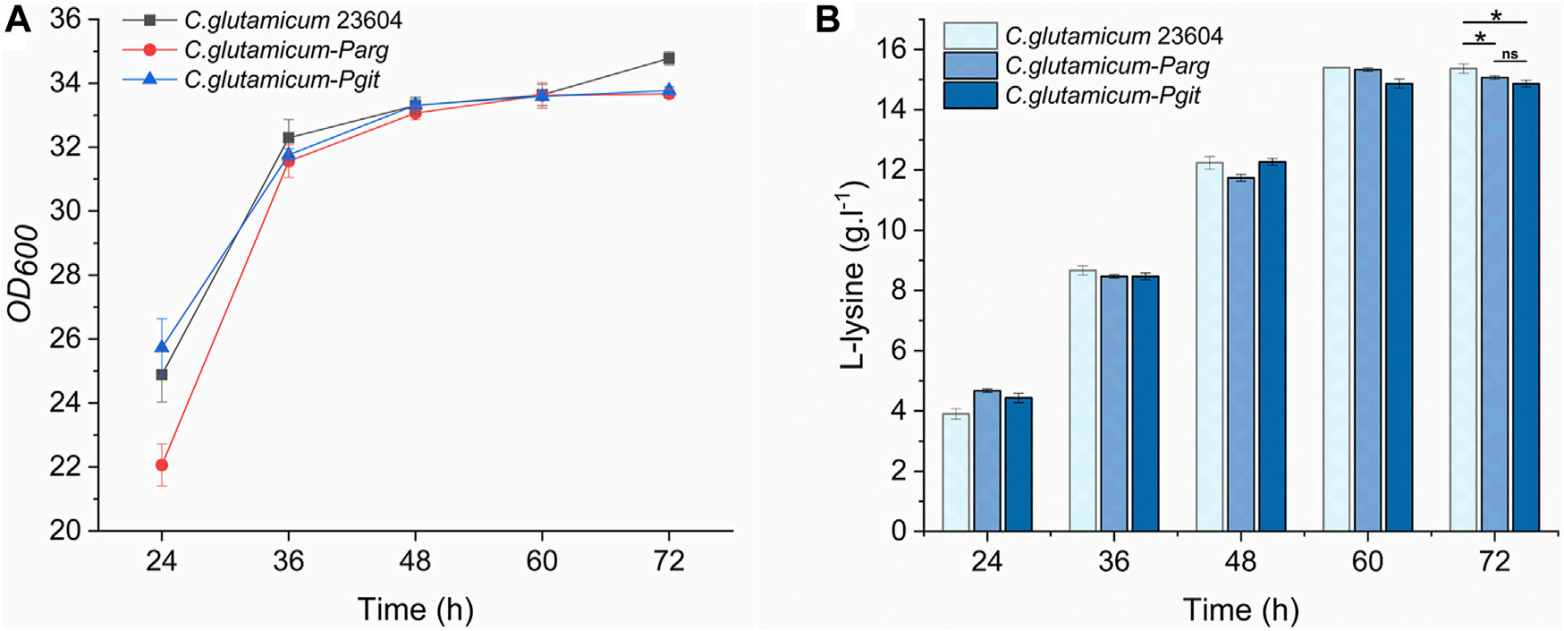
OD_600_ values and L-lysine production in engineered strains of *C. glutamicum-Parg* and *C. glutamicum-Pgit* (fermentation medium). Samples were taken from the fermentation medium of *C. glutamicum-Parg* and *C. glutamicum-Pgit* every 12 h to measure the OD_600_ value **(A)** and L-lysine concentration **(B)**. The *standard errors* are shown as bars.

The knockout vector was constructed using the pK19mobsacB plasmid, and tRNA promoter replacement strains with P*arg* and P*git* promoters were obtained by double-swap homologous recombination. The positive strains, named *C. glutamicum-Parg* and *C. glutamicum-Pgit,* were verified using PCR and electrophoresis (Figures 3A,B). The expression level of tRNA-UUU in the wild type and promoter replacement strains was shown in Supplementary Figure S2, in which the expression level of tRNA-UUU in *C. glutamicum-Parg* was significantly decreased (*p* < 0.01). The fermentation results showed that the production of L-lysine in the final fermentation broth was reduced by 2.3% and 3.1% and that the biomass was reduced by 3.3% and 2.99% in the two promoter replacement strains, *C. glutamicum-Parg* and *C. glutamicum-Pgit*, respectively, compared to those of the wild-type strain *C. glutamicum* 23604 (Figure 4). From the above statistical analysis results, it can be seen that the L-lysine yield of the two promoter replacement strains was significantly different compared to the wild type strain (*p* < 0.05).

### 3.4 Construction of a fluorescent screening system based on rare codons

**FIGURE 5:**
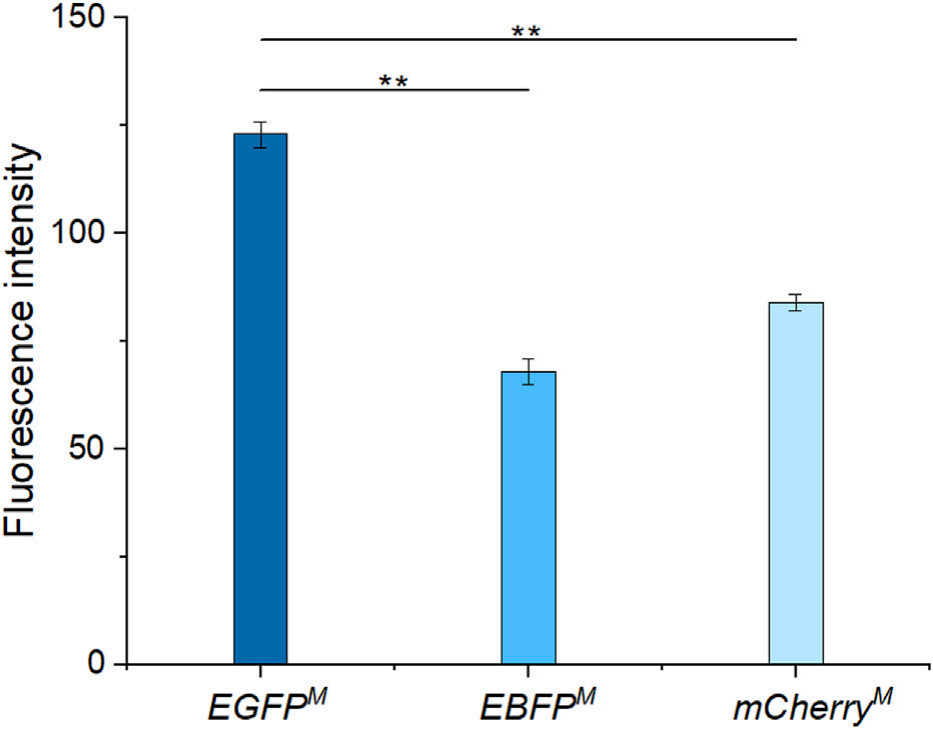
Fluorescence intensities of enhanced green fluorescent protein (EGFP^*M*^), enhanced blue fluorescent protein (EBFP^*M*^), and mCherry^*M*^ *.*

After induction, among the three fluorescent proteins (Figure 5), EGFP^*M*^ had the strongest fluorescence intensity. Therefore, EGFP^*M*^ was finally selected as the fluorescence screening marker. The fluorescent protein expression vector pEC-XK99E-*egfp* ^*M*^ was transformed into a promoter replacement strain, which was identified as positive by PCR and electrophoresis (Figure 3C).

### 3.5 Mutant library constructed using ARTP

**FIGURE 6:**
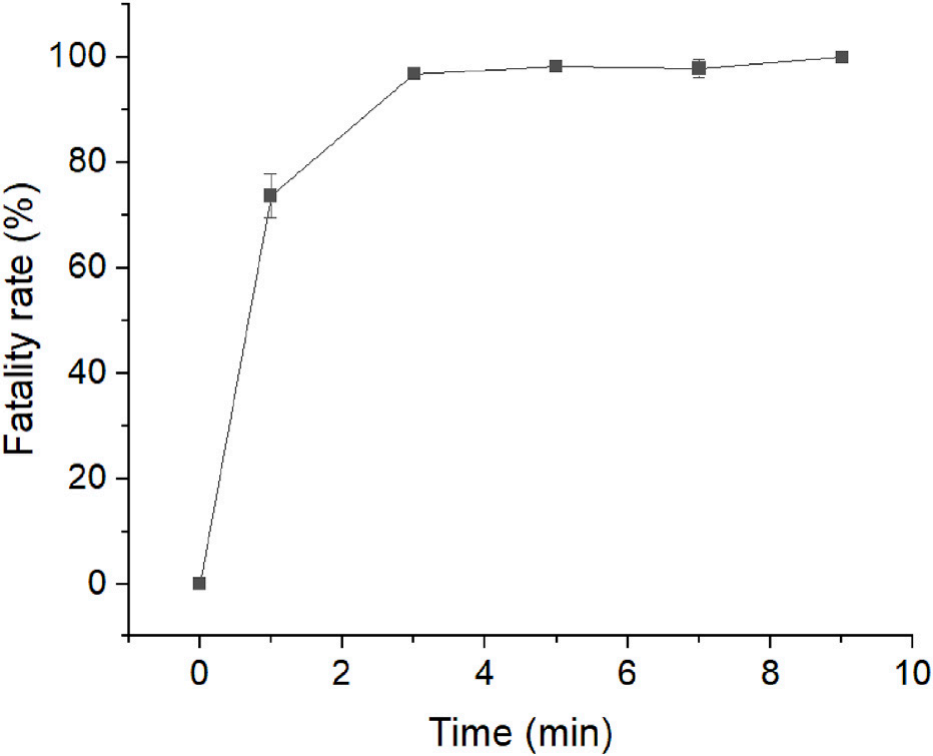
Atmospheric and room-temperature plasma (ARTP) mutagenesis fatality curve.

**FIGURE 7:**
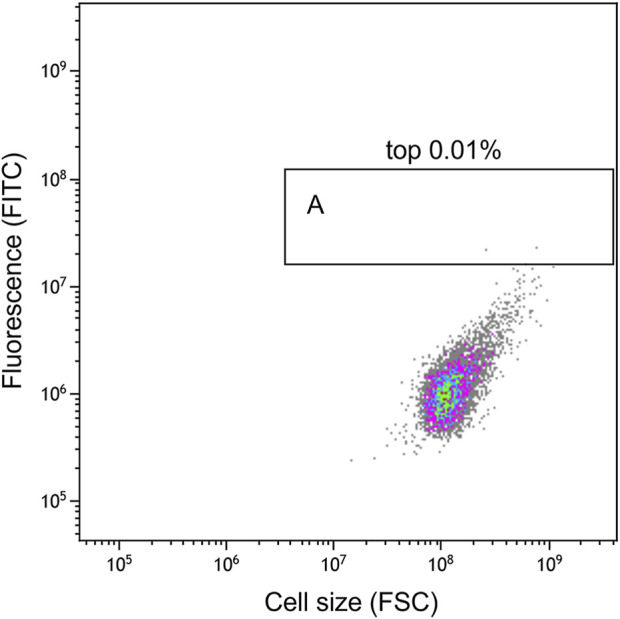
Flow cytometry analysis results. The ordinate FITC shows the fluorescence intensity of EGFP^*M*^

As the flow cytometry sorting system can perform ultra-high-speed sorting and purification of specific cells, it was used to sort the mutants treated with ARTP for 7 min (Figure 6). The ratio of the total number of screened cells to the total cell number was approximately 1/100000. As shown in the analysis and screening results in Figure 7, some cells showed strong fluorescence. Single cells with a stronger fluorescence signal were sorted and inoculated in 96-well plates.

### 3.6 Fermentation of *Corynebacterium glutamicum* mutants

**FIGURE 8:**
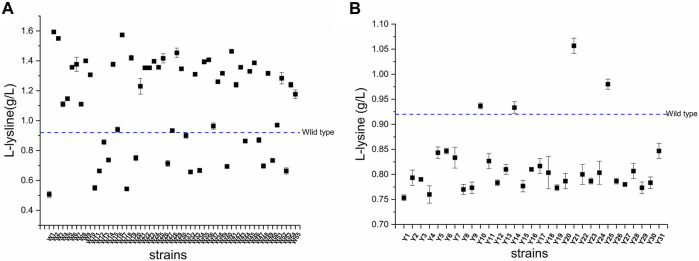
The L-lysine produced by the wild-type and the mutated strains. **(A)** Mutants of *Corynebacterium glutamicum-Parg.* **(B)** Mutants of *Corynebacterium glutamicum-Pgit.*

**FIGURE 9:**
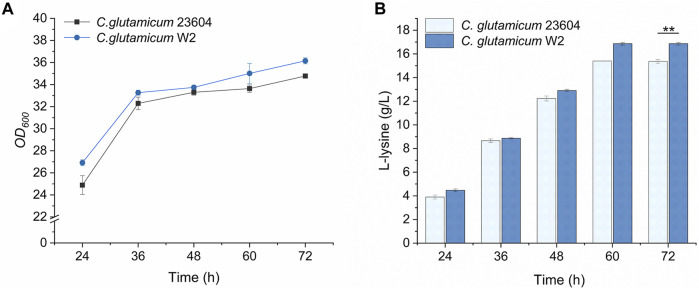
OD_600_ values and L-lysine production in engineered strains of *C. glutamicum* W2 (fermentation medium). During the fermentation process, samples were taken from the fermentation medium every 12 h to measure the OD_600_ value **(A)** and L-lysine concentration **(B)** in *C. glutamicum* 23604 and *C. glutamicum* W2. The *standard errors* are shown as bars.

The results of the fermentation of mutants in 96-well plates are shown in Figure 8. Fifty-five and 31 strains were obtained from the mutants of *C. glutamicum-Parg* and *C. glutamicum-Pgit*, with screening efficiencies of 38/55 and 4/31, respectively. Among them, the screening efficiency of the *C. glutamicum-Parg* mutant reached 69%. Compared with the L-lysine yield of *C. glutamicum* 23604, that of *C. glutamicum* W2 was significantly increased by 9.7% (16.87 g. L^-1^, *p* < 0.05). Furthermore, the biomass of *C. glutamicum* W2 was increased by 3.4%, compared to that of the wild-type *C. glutamicum* 23604 (Figure 9).

## 4 Discussion

Strains of *C. glutamicum* are important industrially as lysine producers. Early amino acid production strains were selected to obtain amino acid high-yielding strains through conventional mutation breeding. With branching in the L-lysine synthesis pathway, feedback inhibition of key enzymes by end products can be lifted by mutation breeding (Ikeda, 2017). The use of amino acid structure analogues is also an important means to breed strains for amino acid production (Kase and Nakayama, 1974). Genetic engineering breeding and metabolic engineering breeding further enhance the L-lysine synthesis pathway by overexpressing its key enzyme-coding genes and increasing the yield by 10%–50% (Jetten et al., 1995; Hartmann et al., 2003; Wang et al., 2015). With the development and application of biosensors including ribose switches, enzymes, and transcription factors, high-throughput screening, which uses micro amounts, is rapid, sensitive, and accurate, has been gradually applied to the screening of amino acid high-yield strains, greatly improving the efficiency of strain screening (Zhang et al., 2018).

High-throughput screening methods using biosensors are used to screen mutant strains that overproduce molecules, such as amino acids and organic acids (Chen et al., 2015; Cress et al., 2015). Binder et al. have developed a biosensor capable of detecting the genetic metabolites of L-lysine in *C. glutamicum* (Binder et al., 2012). This biosensor is based on the transcriptional regulator *lysG* of *C. glutamicum*, which activates the *lysE* promoter to drive the transcription of enhanced yellow fluorescent protein (Cress et al., 2015). High-throughput FACS and ARTP are receiving increasing attention as novel and powerful tools for screening target strains, and this assay, combined with FACS, enables high-throughput screening of large libraries (Mustafi et al., 2012; Della Corte et al., 2020). As a result, ARTP mutagenesis and high-throughput screening techniques were used in this study to increase the L-lysine yield. After the ARTP mutagenesis, L-lysine-overproducing strains were screened using FACS. The fermentation characteristics of mother plants and mutants were evaluated.

Rare codons are being gradually used in the field of strain screening. Yorke Zhang et al. constructed an artificial codon system and found that the growth of *E. coli* was hindered when there were only “artificial genes” containing new codons and no “artificial transport RNA.” Presumably, the lack of transport RNA that could recognize “artificial codons”, resulted in a slower rate of ribosome protein translation in cells, affecting the growth of *E. coli*; this showed that the regulation of the codon system can control the rate of microbial protein synthesis (Zhang et al., 2017). Later, the strategy of using rare codons to screen for amino acid-overproducing bacteria was proposed. Huo et al., for the first time, increased the “threshold” of amino acid concentration required for protein translation by increasing the number of rare codons in the sequence and established a set of amino acid high-yielding strain screening systems by using synthetic GFP rich in natural rare codons, proving that this method was effective in *E. coli* and feasible in *C. glutamicum* (Zheng et al., 2018; Huo et al., 2019). Inspired by this, we constructed an artificial rare codon system for lysine to obtain an over-producer of the amino acid of interest by synonymously substituting its ordinary codon with an artificial rare codon.

In the present study, two artificial rare codon construction strategies were introduced. In the first strategy, the encoded tRNA was directly knocked out of the genome, and surviving strains were not obtained in the second round of screening. We speculate that the loss of this tRNA affects the synthesis of key proteins and the normal growth of *C. glutamicum*. In the second strategy, by replacing the promoter of the tRNA, the transcription frequency was controlled, rendering a common tRNA a “rare tRNA.” Promoter engineering is the primary strategy for precise control of gene expression (Cazier and Blazeck, 2021). Regulation of gene expression can be performed by screening promoters of different intensities and functions. For example, Tan et al. improved the natural *PbrnFE* promoter through promoter engineering and applied it to the dynamic regulation of the expression of idol to promote the synthesis of 4-hydroxyisoleucine in *C. glutamicum* (Tan et al., 2020). Meanwhile, the inducible promoter P*git* and the natural promoter P*arg* were used, the expression of which is induced by glucose, maltose, gluconic acid, and ribose. Thus, it is speculated that the relevant inducing components in the medium will affect the transcription frequency of the tRNA, and the expression of the tRNA can be adjusted by precisely controlling the amount of inducer added to further optimize the screening conditions. Compared with inducible promoters, P*arg* is not affected by inducers, and the experimental results are better. Therefore, tRNA promoter replacement is a viable strategy for constructing rare codons.

In conclusion, the breeding of high-yielding L-lysine strains remains a key goal in the industry, and it may be challenging to further improve the production performance of L-lysine-producing strains. In this study, high-yielding L-lysine strains were obtained by constructing artificial rare codons, combined with ARTP mutagenesis and FACS. This study provides an efficient method for screening a broad range of amino acid high-yielding microbial strains.

## Data Availability

The datasets presented in this study can be found in online repositories. The names of the repository/repositories and accession number(s) can be found below: https://www.ncbi.nlm.nih.gov/, PRJNA974723.

## References

[B1] Binder S. Schendzielorz G. Schendzielorz N. Krumbach K. Hoffmann K. Bott M. (2012). A high-throughput approach to identify genomic variants of bacterial metabolite producers at the single-cell level. Genome Biol. 13, 2012–2013. 10.1186/gb-2012-13-5-r40 PMC3446293 22640862

[B2] Cazier A. P. Blazeck J. (2021). Advances in promoter engineering: Novel applications and predefined transcriptional control. Biotechnol. J. 16, e2100239. 10.1002/biot.202100239 34351706

[B3] Chen W. Zhang S. Jiang P. Yao J. He Y. Chen L. (2015). Design of an ectoine-responsive AraC mutant and its application in metabolic engineering of ectoine biosynthesis. Metab. Eng. 30, 149–155. 10.1016/j.ymben.2015.05.004 26051748

[B4] Cheng J. Chen P. Song A. Wang D. Wang Q. (2018). Expanding lysine industry: Industrial biomanufacturing of lysine and its derivatives. J. Ind. Microbiol. Biotechnol. 45, 719–734. 10.1007/s10295-018-2030-8 29654382

[B5] Cress B. F. Trantas E. A. Ververidis F. Linhardt R. J. Koffas M. A. (2015). Sensitive cells: Enabling tools for static and dynamic control of microbial metabolic pathways. Curr. Opin. Biotechnol. 36, 205–214. 10.1016/j.copbio.2015.09.007 26453934

[B6] Della Corte D. van Beek H. L. Syberg F. Schallmey M. Tobola F. Cormann K. U. (2020). Engineering and application of a biosensor with focused ligand specificity. Nat. Commun. 11, 4851. 10.1038/s41467-020-18400-0 32978386 PMC7519686

[B7] Dittmar K. A. Sorensen M. A. Elf J. Ehrenberg M. Pan T. (2005). Selective charging of tRNA isoacceptors induced by amino-acid starvation. EMBO Rep. 6, 151–157. 10.1038/sj.embor.7400341 15678157 PMC1299251

[B8] Eggeling L. Bott M. (2015). A giant market and a powerful metabolism: L-Lysine provided by *Corynebacterium glutamicum* . Appl. Microbiol. Biotechnol. 99, 3387–3394. 10.1007/s00253-015-6508-2 25761623

[B9] Felix F. Letti L. A. J. Vinicius de Melo Pereira G. Bonfim P. G. B. Soccol V. T. Soccol C. R. (2019). L-Lysine production improvement: A review of the state of the art and patent landscape focusing on strain development and fermentation technologies. Crit. Rev. Biotechnol. 39, 1031–1055. 10.1080/07388551.2019.1663149 31544527

[B10] Geng F. Chen Z. Zheng P. Sun J. Zeng A. P. (2013). Exploring the allosteric mechanism of dihydrodipicolinate synthase by reverse engineering of the allosteric inhibitor binding sites and its application for lysine production. Appl. Microbiol. Biotechnol. 97, 1963–1971. 10.1007/s00253-012-4062-8 22644522

[B11] Guimaraes J. C. Mittal N. Gnann A. Jedlinski D. Riba A. Buczak K. (2020). A rare codon-based translational program of cell proliferation. Genome Biol. 21, 44. 10.1186/s13059-020-1943-5 32102681 PMC7045563

[B12] Hamid S. N. I. N. Abdullah M. F. Zakaria Z. Yusof S. J. H. M. Abdullah R. (2016). Formulation of fish feed with optimum protein-bound lysine for African catfish (*Clarias gariepinus*) fingerlings. Procedia Eng. 148, 361–369. 10.1016/j.proeng.2016.06.468

[B13] Hartmann M. Tauch A. Eggeling L. Bathe B. Mockel B. Puhler A. (2003). Identification and characterization of the last two unknown genes, *dapC* and *dapF*, in the succinylase branch of the L-lysine biosynthesis of *Corynebacterium glutamicum* . J. Biotechnol. 104, 199–211. 10.1016/s0168-1656(03)00156-1 12948639

[B14] Huo Y. X. Zheng B. Wang N. Yang Y. Liang X. Ma X. (2019). Identifying amino acid overproducers using rare-codon-rich markers. J. Vis. Exp. 148. 10.3791/59331 31282885

[B15] Ikeda M. (2017). Lysine fermentation: History and genome breeding. Adv. Biochem. Eng. Biotechnol. 159, 73–102. 10.1007/10_2016_27 27832296

[B16] Jetten M. S. Follettie M. T. Sinskey A. J. (1995). Effect of different levels of aspartokinase on the lysine production by *Corynebacterium lactofermentum* . Appl. Microbiol. Biotechnol. 43, 76–82. 10.1007/BF00170626 7766138

[B17] Kase H. Nakayama K. (1974). Mechanism ofl-threonine andl-lysine production by analog-resistant mutants of*Corynebactemum glutamicum* . Corynebactemum glutamicum Agric. Biol. Chem. 38, 993–1000. 10.1080/00021369.1974.10861280

[B18] Li Y. Wei H. Wang T. Xu Q. Zhang C. Fan X. (2017). Current status on metabolic engineering for the production of L-aspartate family amino acids and derivatives. Bioresour. Technol. 245, 1588–1602. 10.1016/j.biortech.2017.05.145 28579173

[B19] Liu J. Xu J. Z. Rao Z. M. Zhang W. G. (2022). Industrial production of L-lysine in *Corynebacterium glutamicum*: Progress and prospects. Microbiol. Res. 262, 127101. 10.1016/j.micres.2022.127101 35803058

[B20] Mohsin M. Ahmad A. Iqbal M. (2015). FRET-based genetically-encoded sensors for quantitative monitoring of metabolites. Biotechnol. Lett. 37, 1919–1928. 10.1007/s10529-015-1873-6 26184603

[B21] Mustafi N. Grunberger A. Kohlheyer D. Bott M. Frunzke J. (2012). The development and application of a single-cell biosensor for the detection of L-methionine and branched-chain amino acids. Metab. Eng. 14, 449–457. 10.1016/j.ymben.2012.02.002 22583745

[B22] Nakamori S. (2017). Early history of the breeding of amino acid-producing strains. Adv. Biochem. Eng. Biotechnol. 159, 35–53. 10.1007/10_2016_25 28058453

[B23] Nguyen L. Davis D. A. (2016). Comparison of crystalline lysine and intact lysine used as a supplement in practical diets of channel catfish (*Ictalurus punctatus*) and Nile tilapia (*Oreochromis niloticus*). Aquaculture 464, 331–339. 10.1016/j.aquaculture.2016.07.005

[B24] Piwowarek K. Lipińska E. Hać-Szymańczuk E. Kieliszek M. Kot A. M. (2020). Sequencing and analysis of the genome of *Propionibacterium freudenreichii* T82 strain: Importance for industry. Biomolecules 10, 348. 10.3390/biom10020348 32102319 PMC7072396

[B25] Subramaniam A. R. Zid B. M. O'Shea E. K. (2014). An integrated approach reveals regulatory controls on bacterial translation elongation. Cell. 159, 1200–1211. 10.1016/j.cell.2014.10.043 25416955 PMC4243059

[B26] Tan S. Shi F. Liu H. Yu X. Wei S. Fan Z. (2020). Dynamic control of 4-hydroxyisoleucine biosynthesis by modified L-Isoleucine biosensor in recombinant *Corynebacterium glutamicum* . ACS Synth. Biol. 9, 2378–2389. 10.1021/acssynbio.0c00127 32813974

[B27] Wang J. Gao D. Yu X. Li W. Qi Q. (2015). Evolution of a chimeric aspartate kinase for L-lysine production using a synthetic RNA device. Appl. Microbiol. Biotechnol. 99, 8527–8536. 10.1007/s00253-015-6615-0 25935345

[B28] Wang J. Gao C. Chen X. Liu L. (2021). Expanding the lysine industry: Biotechnological production of L-lysine and its derivatives. Adv. Appl. Microbiol. 115, 1–33. 10.1016/bs.aambs.2021.02.001 34140131

[B29] Xiao J. Wang D. Wang L. Jiang Y. Xue L. Sui S. (2020). Increasing L-lysine production in *Corynebacterium glutamicum* by engineering amino acid transporters. Amino Acids 52, 1363–1374. 10.1007/s00726-020-02893-6 33021685

[B30] Xu J. Z. Yu H. B. Han M. Liu L. M. Zhang W. G. (2019). Metabolic engineering of glucose uptake systems in *Corynebacterium glutamicum* for improving the efficiency of L-lysine production. J. Ind. Microbiol. Biotechnol. 46, 937–949. 10.1007/s10295-019-02170-w 30937555

[B31] Yu C. H. Dang Y. Zhou Z. Wu C. Zhao F. Sachs M. S. (2015). Codon usage influences the local rate of translation elongation to regulate co-translational protein folding. Mol. Cell. 59, 744–754. 10.1016/j.molcel.2015.07.018 26321254 PMC4561030

[B32] Zhang X. Zhang X. Li H. Wang L. Zhang C. Xing X. (2014). Atmospheric and room temperature plasma (ARTP) as a new powerful mutagenesis tool. Appl. Microbiol. Biotechnol. 98, 5387–5396. 10.1007/s00253-014-5755-y 24769904

[B33] Zhang Y. Ptacin J. L. Fischer E. C. Aerni H. R. Caffaro C. E. San Jose K. (2017). A semi-synthetic organism that stores and retrieves increased genetic information. Nature 551, 644–647. 10.1038/nature24659 29189780 PMC5796663

[B34] Zhang X. Zhang X. Xu G. Zhang X. Shi J. Xu Z. (2018). Integration of ARTP mutagenesis with biosensor-mediated high-throughput screening to improve L-serine yield in *Corynebacterium glutamicum* . Appl. Microbiol. Biotechnol. 102, 5939–5951. 10.1007/s00253-018-9025-2 29725721

[B35] Zheng B. Ma X. Wang N. Ding T. Guo L. Zhang X. (2018). Utilization of rare codon-rich markers for screening amino acid overproducers. Nat. Commun. 9, 3616. 10.1038/s41467-018-05830-0 30190534 PMC6127279

