# Supplementary material for: Design and application of artificial rare L-lysine codons in Corynebacterium glutamicum
Source: Front Bioeng Biotechnol. 2023 May 30;11:1194511. doi: 10.3389/fbioe.2023.1194511 (PMC10268032; doi:10.3389/fbioe.2023.1194511)
Supplement: Supplementary file 1 [file DataSheet1.pdf]

Supplementary material

Design and application of artificial rare L-lysine codons in *Corynebacterium glutamicum*

Cuiping Yang, Zehao Peng, Lu Yang, Bowen Du, Chuanzhuang Guo, Songsen Sui, Jianbin Wang, Junlin Li, Junqing Wang* and Nan Li*

*** Correspondence:**
Junqing Wang and Nan Li
E-mail: wjqtt.6082@163.com (Junqing Wang), and linan1166@mail.tust.edu.cn (Nan Li)

1 Materials and methods of RT-qPCR

C. glutamicum-Parg, *C. glutamicum-Pgit* and wild strain *C. glutamicum* 23604 were inoculated in LBG medium at 30 °C, 200 r/min until OD600 was 1.0. Total RNA was extracted using RNAprep PureCell/Bacteria Kit (Tiangen, China), and then reverse transcribed using HiScript® II Q RTSuperMix for qPCR (+gDNA wiper) (Vazyme Biotech, Nanjing, China) kit. The samples were prepared using ChamQTM UniversalSYBR qPCR Master Mix (Vazyme Biotech, Nanjing, China). Then RT-qPCR analysis was performed using a fluorescent quantitative PCR instrument (Applied Biosystems, USA), and 16s rRNA genes were selected as internal reference genes for quantification. Three biological replicates were performed, and the average threshold cycle was calculated. Finally, the relative expression levels of tRNA-UUU were calculated. The RT-qPCR primers used in this study are shown in Supplementary Table 1.

Tables

Supplementary Table 1 Primers used in RT-qPCR.

Primer	Sequence (5′→3′)
S1	TTTGGTGGATGAGTGGCGAA
S2	GACGCCTTGGTAGGCCATTA
S3	GGGCCTATAGCTCAGTTGGTAGAG
S4	GTGGGCCCATTGGGACTC

Figures

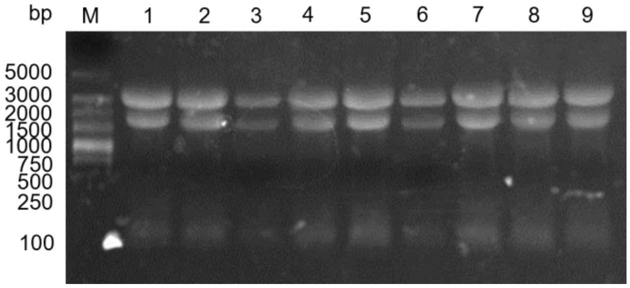

Supplementary Figure 1 Gel electrophoresis of total RNA. 1-3, *C. glutamicum* 23604, 4-6, *C. glutamicum-Pgit*, 7-9, *C. glutamicum-Parg*.

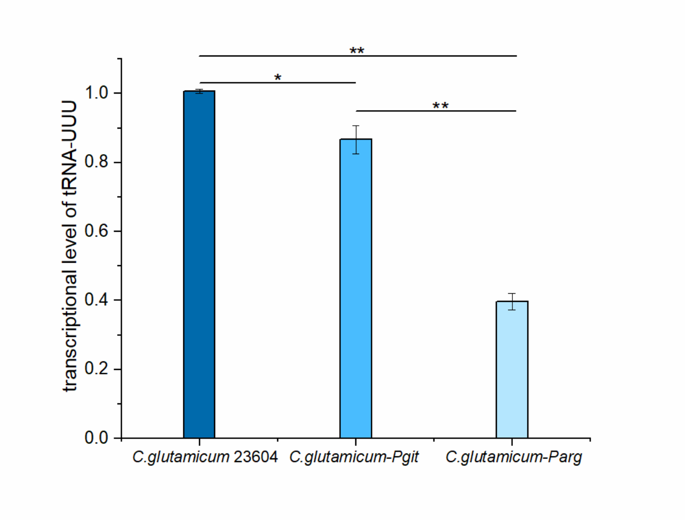

Supplementary Figure 2 Transcriptional level of tRNA-UUU.
